# Semi-Immersive Virtual Reality Exercise Therapy for Upper Limb Rehabilitation in Patients With Spinal Cord Injury Using the Leap Motion Controller

**DOI:** 10.7759/cureus.52261

**Published:** 2024-01-14

**Authors:** Moaied Mohamed Ahmed Al Nattah, Simone Tiberti, Leandro Segaletti

**Affiliations:** 1 Physical and Rehabilitation Medicine, University of Rome, Sapienza, Rome, ITA; 2 Spinal Unit, C.T.O. Andrea Alesini di Roma, Rome, ITA

**Keywords:** physiatrist, upper limbs, physical exercise, muscle weakness, occupational therapy, physical medicine and rehabilitation, unity 3d, leap motion controller, semi-immersive virtual reality, spinal cord injury

## Abstract

In this article, we present a case study involving a patient with spinal cord injury (SCI), resulting in tetraplegia and subsequent loss of upper limb function. The subject of our study was a 23-year-old woman with incomplete tetraplegia stemming from a cervical spinal cord injury. Our primary objective was to enhance hand function and grip strength. Throughout the intervention, we observed substantial enhancements in hand function, range of motion, and muscle power. Notably, the patient exhibited a favorable response to the therapy, demonstrating commendable adherence and active participation.

To create an optimal training environment tailored to the patient's needs, we employed the Unity 3D game engine in conjunction with a Leap Motion controller sensor. This combination facilitated the development of a semi-immersive virtual training environment. The utilization of this technology aimed to simulate a conducive training atmosphere for the rehabilitation of hand function.

Based on our study outcomes, we advocate for the incorporation of leap motion-related exercises in the treatment of hand functional loss and weakness. The promising results observed in this case study prompt the recommendation for further large-scale studies to validate and substantiate our findings. Such investigations would contribute to the establishment of evidence-based practices and enhance the understanding of the efficacy of Leap Motion technology in addressing upper limb impairments associated with spinal cord injuries.

## Introduction

The advancement of technology in the field of rehabilitation has paved the way for innovative approaches to address the complex challenges faced by individuals with spinal cord lesions, particularly those with tetraplegia. Upper-limb rehabilitation plays a crucial role in enhancing functional independence and quality of life for such patients. This case report delves into the application of Leap Motion Controller technology as a novel intervention for upper limb rehabilitation in a tetraplegic patient with a spinal cord lesion.

Tetraplegia, resulting from spinal cord injuries, presents profound physical limitations, often impacting the individual's ability to perform the basic activities of daily living. Traditional rehabilitation approaches are frequently met with unique challenges in this population, necessitating the exploration of cutting-edge technologies to optimize therapeutic outcomes. The Leap Motion controller, a motion tracking device known for its precision and versatility, offers a promising avenue for tailored upper limb rehabilitation in these individuals.

The Leap Motion controller operates by capturing and interpreting intricate hand and finger movements in three-dimensional space through the use of infrared sensors. This technology enables healthcare practitioners to engage with digital interfaces in a natural and intuitive manner, opening avenues for innovative applications in diagnostics, treatment planning, and rehabilitation. The intersection of leap motion and medicine represent a convergence of advanced computing capabilities with the intricacies of human anatomy and physiology.

The Leap Motion controller has garnered attention for its potential applications across diverse domains, ranging from virtual reality and gaming to medical and rehabilitation settings. Its ability to track intricate hand movements in real-time provides a new dimension to user interface design, offering an intuitive and immersive interaction experience. 

The focus of this exploration is to unravel how Leap Motion technology can be harnessed to address the specific challenges associated with upper limb impairments. From gamified exercises that promote patient engagement to precise tracking of fine motor movements essential for rehabilitation, Leap Motion holds the potential to transform traditional rehabilitation paradigms. By seamlessly integrating technology into the rehabilitation process, Leap Motion not only enhances the efficacy of therapeutic interventions but also provides a platform for personalized and patient-centric approaches.

This case report seeks to provide a comprehensive exploration of the utilization of Leap Motion technology in the rehabilitation journey of a tetraplegic patient. Through an in-depth examination of the patient's clinical history, the specific nature of their spinal cord lesion, and the targeted interventions using Leap Motion, this research aims to contribute valuable insights into the feasibility, efficacy, and potential benefits of this technological approach. The overarching goal is to bridge the gap in our understanding of upper limb rehabilitation strategies for tetraplegic individuals and to inform future advancements in the field of neurorehabilitation.

## Case presentation

The patient

A 23-year-old female patient, presenting with tetraplegia at the C5 level and classified as ASIA impairment scale (A.I.S.) incomplete D, Spinal Cord Independence Measure (SCIM) 13, was admitted to the emergency room following a car accident. The patient sustained cervical spine compression, mild head trauma, cerebellar contusion, and subdural hematoma. A CT scan revealed a type C fracture of a cervical vertebra with posterior dislocation and spinal cord compression. Subsequent treatment at the neurosurgery department included a neurosurgical intervention involving corpectomy C5, plating C4-C6, and posterior arthrodesis C4-C6. The patient was fitted with a cervical collar.

Throughout her hospitalization, the patient received medications, including laroxyl, ansiolin, diazepam, gutron, lyrica, stilnox, brufen, and paracetamol, with variable doses based on her clinical condition. The Sollerman test was conducted at T0 (7 days after initiating VR treatment) and at T1 (after 70 days), alongside passive muscular exercises during hospitalization. At T0, the patient scored 52/80 with her right hand and 60/80 with her left hand on the Sollerman test. At T1, notable improvement was observed, with scores of 73/80 for the right hand and 75/80 for the left hand.

**Figure 1:**
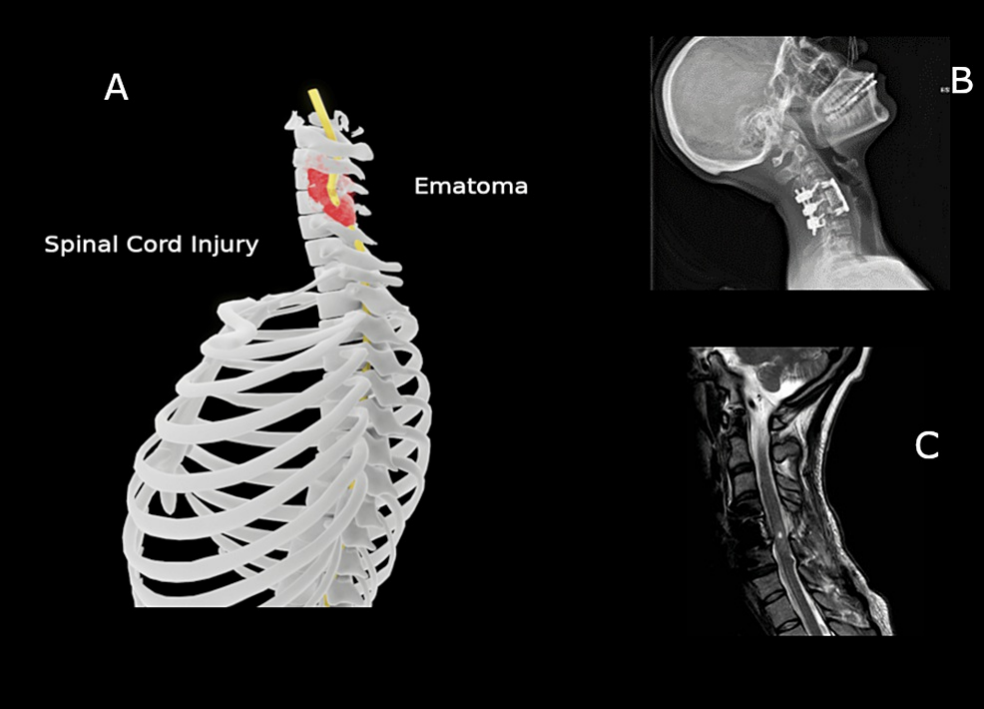
Spinal cord injury and the patient's cervical spine imaging A: An illustration of cervical spinal cord injury. The 3D graphic was made by the author, Dr. Al Nattah. B: X-ray showing surgical operation of cervical vertebral column stabilization. C: Magnetic resonance imaging (MRI) showing cervical spinal cord injury SCI. The permission was obtained from the patient.

**Figure 2:**
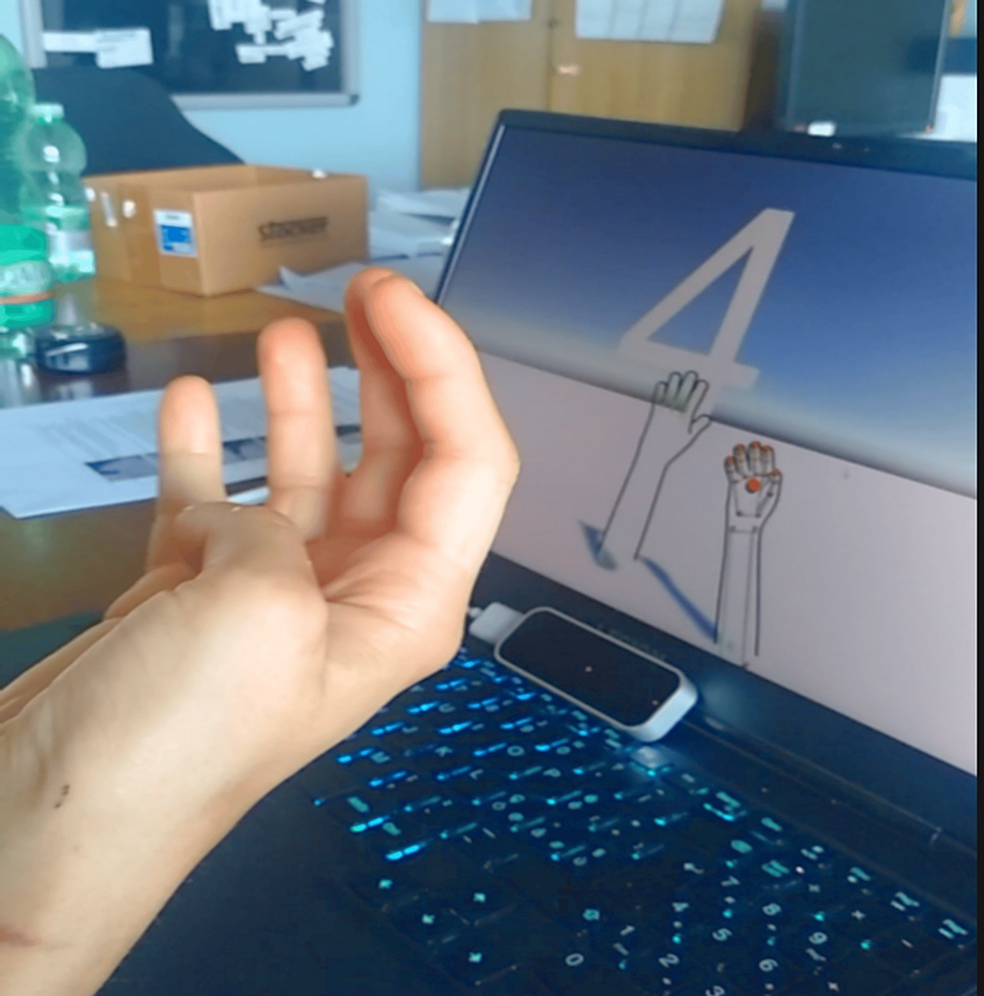
Leap motion hand tracking Finger extension before therapy.

**Figure 3:**
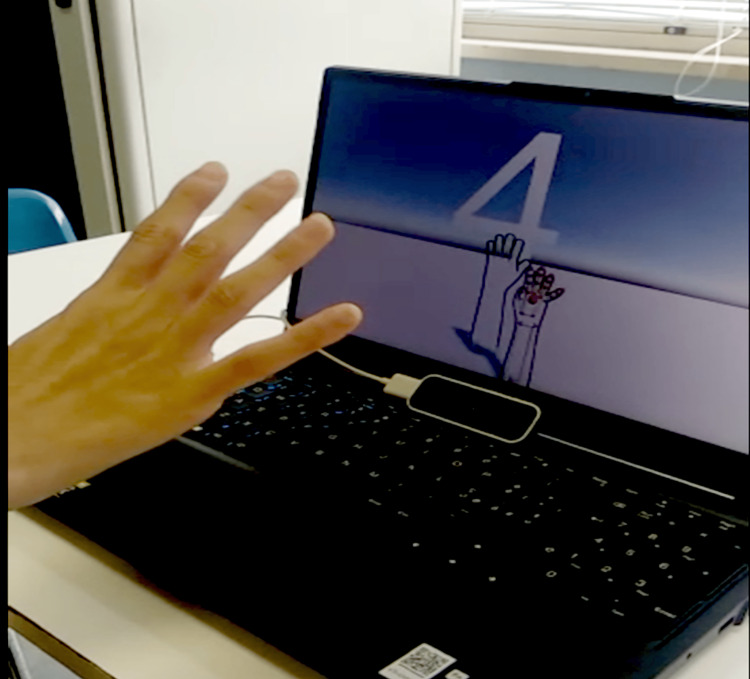
Leap motion hand tracking Finger extension after therapy.

The Medical Research Council (MRC) score for the right upper limb increased from 2/5 at admission to 4/5 at discharge. Further details and the timeline of leap motion therapy can be found in Figures 1-3 and Table 1, respectively.

**Table 1:** Timeline of upper limb leap motion therapy

2022-06-13	First Sollerman Test
2022-06-14	First Recorded Video
2022-06-27	The Second time Recorded Video
2022-08-22	Final Sollerman Test

VR Leap Motion therapy was administered to a tetraplegic patient experiencing impaired upper limb functionality, and its effectiveness was assessed using the ASIA and SCIM scales. The patient underwent the Sollerman test to evaluate residual functional capacity, displaying improvement over an approximately three-month period through therapeutic exercises. Various VR applications and video games were employed to facilitate upper-limb movement within an interactive virtual environment. Auditory feedback was integrated to enhance the immersive nature of the experience, fostering patient engagement and compliance during exercises.

The study encompasses several objectives: 1) To explore the impact of leap motion exercises on patients with spinal cord injuries. 2) To assess the efficacy of this particular treatment modality. 3) To analyze the overall outcomes of the therapy. 4) To ascertain the potential benefits of the treatment for hand rehabilitation in patients with tetraplegia.

Virtual reality exercise therapy setting

The patient was situated in front of a computer screen, with the Leap Motion sensor strategically positioned to detect hand movements. The sensor's placement was adjustable to ensure precise tracking. Prior to commencing any exercises, a preliminary test was conducted to verify the functionality and positioning of the sensor. In instances of malfunction, the device could be reset and repositioned.

Several video game exercises were incorporated, each offering varying levels of difficulty tailored to the specific functionality of the patient's upper limbs. One exercise involved counting from 1 to 5 using fingers, while another required the execution of pronation and supination movements with corresponding visual feedback displayed on the screen. Auditory feedback signals were employed to indicate the successful execution of supination or pronation or the correct performance of the activity.

The kinematics of upper limb movements were meticulously analyzed, encompassing the recording of body segment position, orientation, and joint angles at distinct time points. In essence, the overarching objective of these interactive exercises was to enhance upper-limb function through targeted physical activities and muscle training.

Creation of the "counting with fingers" game

In order to detect the right- or left-hand finger extension, a finger extension detector was integrated. This game component is designed to discern finger flexion and extension, enabling the execution of scripts upon activation or deactivation. Additionally, a feedback sound is incorporated to signal the successful completion of an action, such as finger extension or flexion.

Creation of the pronation and supination, wrist flexion, and extension game

The integration of a palm detector enables players to detect their palm's position in either pronation or supination, as well as discern whether their wrist is in a state of flexion or extension. A sound cue signifies the successful completion of a task.

Creation of the hand grasp game

**Figure 4:**
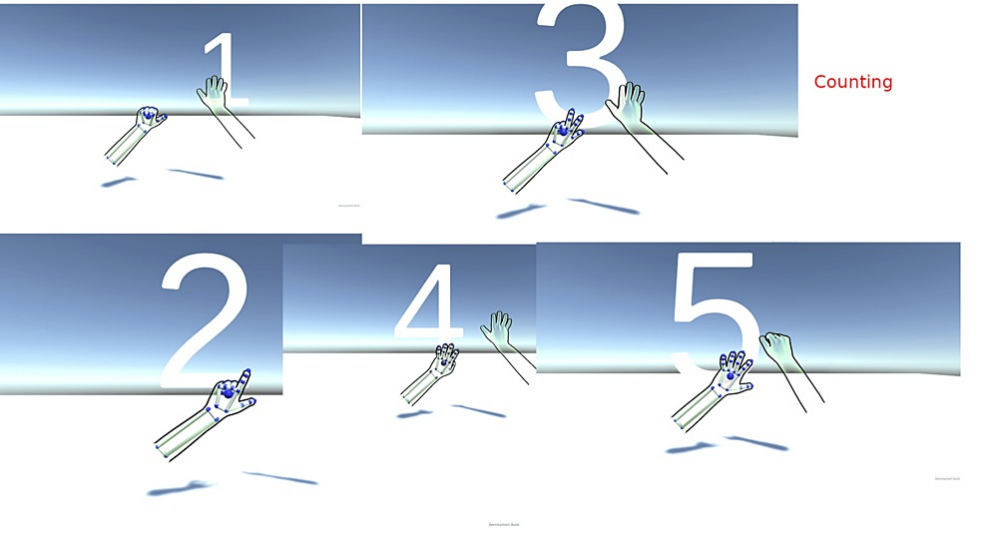
Counting exercises with leap motion These images are screenshots of the game made by the author while the patient does hand exercises.

**Figure 5:**
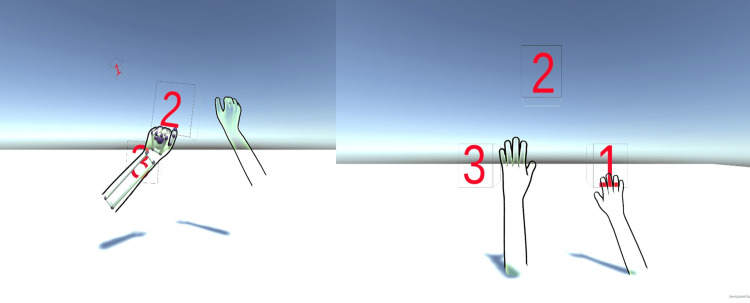
Hand-grasping exercises These images are screenshots of the game made by the author while the patient does hand exercises.

The incorporation of a pinch detector into the right- or left-hand model is accompanied by the introduction of a cube within the scene for interactive purposes. The cube features a rigid body component and an interaction behavior component, and sound feedback is triggered upon successful grasping of the cube. Subsequent adjustments to the scene were made, and multiple experiments were conducted to assess the game (Figures 4, 5) and (Videos 1-3).

**Video 1:** Index finger extension before therapy The patient has limited index finger extension before therapy.

**Video 2:** Index finger extension after the therapy The patient has full index finger extension after the therapy.

**Video 3:** Hand grip exercise Hand grip before the therapy.

Patient perspective

The patient was requested to contribute her data for research purposes, a request to which she willingly consented. The patient demonstrated commendable compliance with the therapy and conveyed a positive attitude toward the treatment. At the conclusion of the therapy, a questionnaire was administered to the patient. The patient's responses indicated an excellent experience, positive outcomes, and a high level of satisfaction with Leap Motion therapy.

## Discussion

**Figure 6:**
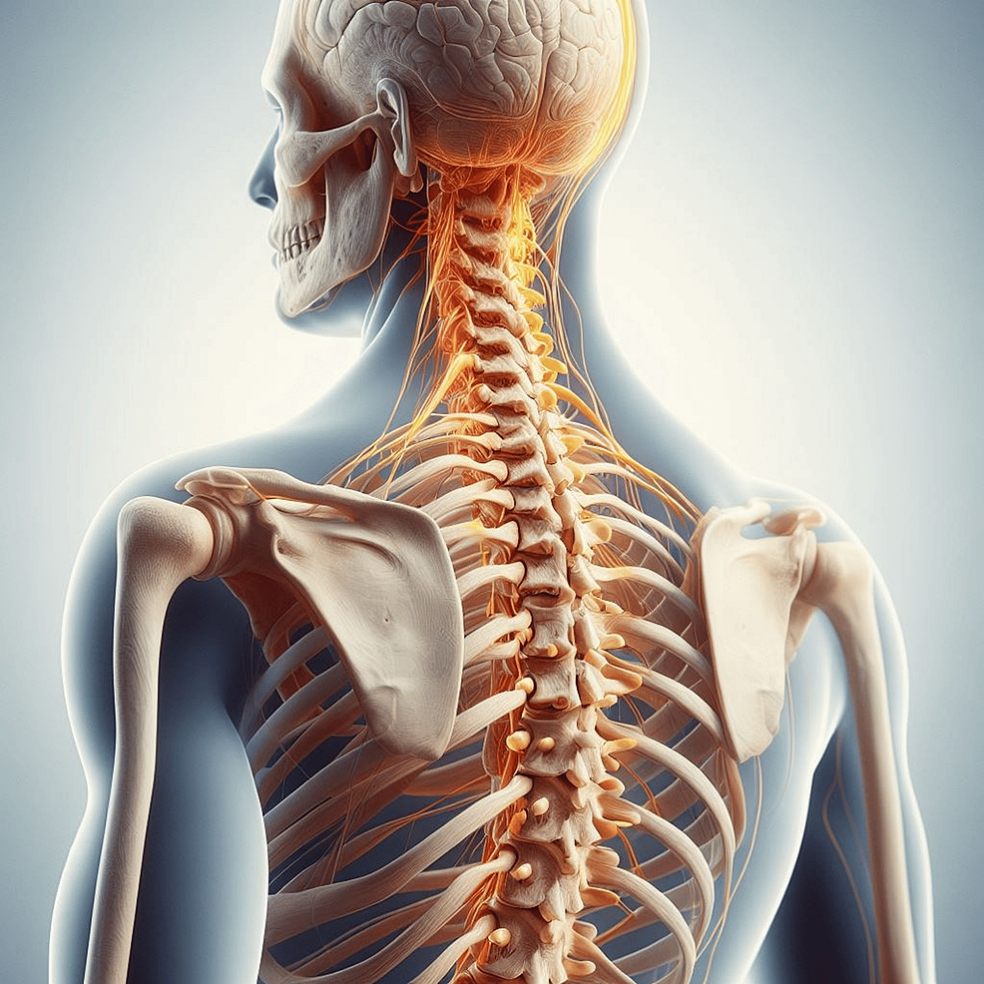
Spine anatomy Vertebral column and central nervous system. Created by artificial intelligence (AI), image creator.

**Figure 7:**
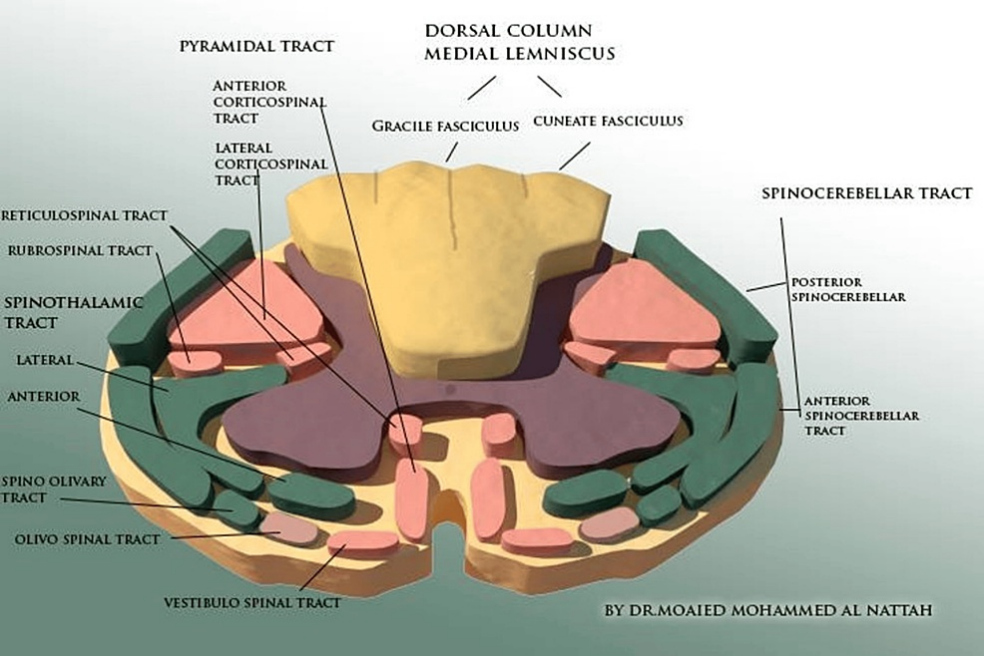
Spinal cord cross section and nerve pathways Created by Dr. Al Nattah, the author of this case report

The spinal cord is part of the central nervous system (CNS) that extends caudally and is protected by the bony structures of the vertebral column. It is covered by the three membranes of the CNS, namely the dura mater, arachnoid, and innermost pia mater, as mentioned by Nógrádi et al. [1]. The spinal cord is a continuation of the brainstem, beginning at the foramen magnum and traversing the vertebral foramen to the lower border of the first lumbar vertebra (L1) in adults (Figure 6) and the lower border of the second or upper border of the third lumbar vertebra (L2/L3) in growing children. The spinal cord contains numerous groups of nerve fibers responsible for carrying sensory and motor stimuli to and from the periphery, collectively called the ascending and descending tracts of the spinal cord, as referred to by Snell et al. [2] (Figure 7). Spinal cord injuries due to traumatic fractures can lead to persistent neurological deficits such as muscle weakness. Closed reduction of the cervical spine is a common treatment method for acute subluxations or dislocations aiming to restore spinal alignment and stability, according to NICE Guideline No. 41 [3] (Figure 1). The complications associated with immobility include a limited range of motion, and in some cases, the position of the joint may become permanently fixed, leading to contracture deformities, as emphasized by Born et al. [4]. Range-of-motion and resistive exercises, upright positioning, and strengthening exercises should be employed as soon as possible through physiotherapy and occupational therapy assessment to maintain muscle function, mobilization, and training with mobilization aids such as crutches or frames. Basic activities of daily living should also be supported through training or aids, according to NICE Guideline No. 211 [5].

Semi-immersive VR is a desktop VR development and includes additional devices such as data gloves or other tracking sensors. It keeps the simplicity of the desktop VR system but with a high level of immersion. For building a semi-immersive system, the requirements are a computer, tracking sensors, a leap motion, and a user interface, as mentioned by Alqahtani et al. [6].

Unity 3D is a game engine that makes creators create games and simulations of real-life situations easily, as expressed by Creighton et al. [7].

The integration of leap motion technology with upper limb rehabilitation holds immense potential for enhancing patient engagement and promoting faster recovery. In the study conducted by Cortés-Pérez et al. [8], it was observed that the use of a leap motion controller (LMC) resulted in enhanced mobility of the upper extremities and improved performance in tasks related to upper extremity mobility in stroke patients. Similarly, in individuals with non-acute central nervous system disease (CNSD), LMC was found to increase grip strength (GS) and gross motor dexterity (GMD) of the most affected upper extremity, as well as improving fine motor dexterity (FMD) when used in conjunction with conventional therapy (CT).

Also, Leap Motion technology has demonstrated its utility in upper limb rehabilitation for individuals with spinal cord lesions, as well as in the broader spectrum of neurologic and orthopedic conditions characterized by upper limb impairments. Here are some examples:

Leap motion has been employed as a tool to assess the severity of bradykinesia (slowness of movement) and monitor it during surgical interventions, as documented by Wu et al. [9]. It has also been utilized in clinical examinations for Parkinson's disease, demonstrating favorable outcomes for certain movements, as reported by Butt et al. [10]. However, its widespread application necessitates further development to improve the performance of the Leap Motion Controller, as emphasized by Garcia-Agundez et al. [11]. Leap motion has additionally been employed in upper limb therapy for Parkinson's disease, resulting in improved upper limb function, as demonstrated by Cikajlo et al. [12].

A systematic review by Tarakci et al. [13] identified the benefits of leap motion therapy for adolescents and children with disabilities such as juvenile idiopathic arthritis (JIA), cerebral palsy (CP), and brachial plexus birth injury (BPBI), which are the most common disorders that cause upper extremity impairments in children and adolescents. Nevertheless, the study faced limitations such as small sample sizes and the quality of the included studies. The comparisons between leap motion controller-based training (LMCBT) and conventional treatment groups showed similar results in all parameters in all disease groups. Wang et al. [14] showcased upper limb improvement with Leap Motion in stroke patients, and Leap Motion-based virtual reality training could facilitate cortical reorganization and might facilitate the motor function recovery of an affected upper limb in patients who had experienced a subacute stroke.

Moreover, Leap Motion has served as a non-contact tool for user interface control in a contactless, sterile environment, particularly in operating rooms, as highlighted by Alvarez-Lopez et al. [15]. It has also shown enhanced upper limb function for multiple sclerosis patients, and in the experimental group compared to the control group, significant improvements were observed in the post-treatment assessment for coordination, speed of movements, and fine and gross upper limb dexterity. Also, significant results were found in the follow-up in coordination, speed of movements, and fine and gross for the more affected side, as demonstrated by Cuesta-Gómez et al. [16]. However, findings regarding distal radial fractures were inconclusive, according to Arora et al. [17].

These virtual reality exercises are characterized by their simplicity and ease of execution within a virtual environment. The patient engages in wrist and finger flexion, cube grasping, and arm movement facilitated by the virtual environment known as WoW (Windows on the World). In this setting, the computer screen functions as a window displaying a virtual hand that replicates the patient's hand movements. The Leap Motion controller accurately tracks the patient's hand motions, projecting them onto the screen. The WoW environment allows for customization, enabling the inclusion of diverse objects, auditory stimuli, and visual effects. In this study, elements such as boxes, numerical cues, and auditory feedback were incorporated to enhance the immersive and interactive nature of the experience. This augmentation serves to provide the patient with real-time feedback, enabling them to respond to their actions.

Each iteration of the exercise requires the patient to perform the task repeatedly for approximately one minute. The exercise duration spans from 30 minutes to one hour, contingent upon the patient's specific condition. The therapeutic intervention is administered daily, five days a week. In instances where the patient experiences physical discomfort or deviates from normal bowel habits, leading to an inability to perform the exercises, the therapy is temporarily halted until the patient's condition stabilizes.

Results indicate that virtual reality therapy has demonstrated efficacy in enhancing the range of motion, mitigating the risk of soft tissue contracture following hand disuse, and expediting the recovery of hand function. This therapeutic approach represents a promising avenue for improving rehabilitation outcomes for patients with impaired hand function.

This training protocol is classified as a form of physical exercise with the potential to reduce hospitalization durations while concurrently enhancing patient autonomy and proficiency in daily living activities. The adaptability of the Leap Motion equipment facilitates the extension of hand exercises to the patient's home environment. The development of Leap Motion applications necessitates expertise in C# programming and a comprehension of 3D graphics. Crucial components include the creation of models, scene preparation, and the seamless integration of graphics with coding within the Unity 3D environment, aligned with a logical understanding of the patient's condition.

Proficiency in the Unity 3D environment is facilitated by Leap Motion's Software Development Kit (SDK), offering access to pre-built projects, functions, methods, models, and other essential data to streamline project development. The game scene encompasses diverse elements such as models, classes, game components, and scripts. The musculoskeletal benefits of exercising include heightened muscle tone and strength, diminished soft tissue stiffness, and the initiation of nerve rewiring, as noted by Hicks et al. [18] and Born et al. [4]. Notably, a young patient subjected to early VR treatment within three months post-tetraplegic injury, featuring neurological bladder symptoms, demonstrated remarkable improvements in a relatively brief timeframe.

The game scene is intentionally designed with minimal distractions, prioritizing the patient's focus on hand exercises without interference from music or visual effects. The exercises, characterized by repetition, simplicity, muscle/joint orientation, and entertainment, are meticulously supervised to ensure patient safety while aligning with their endurance levels. If the patient feels capable, they may continue exercising, with the option to rest when needed.

In theory, any game involving finger and hand movements can be developed, with the utilization of inverse kinematics enabling elbow and shoulder movement manipulation by controlling the hands. This form of kinematics, termed "inverse," allows for distal part movement to influence proximal parts, in contrast to "forward" kinematics, where proximal part movement dictates the position and orientation of distal parts.

The present case report on virtual reality (VR) in rehabilitation includes a notable constraint related to the patient population. It is important to note that not every patient can achieve favorable outcomes when utilizing VR technology for rehabilitation due to specific prerequisites such as residual functional capacity and muscle power required to perform exercises and facilitate recovery. Therefore, if an older patient with diminished functional residual capacity is involved, the extent of their recuperation might be limited. Additionally, limitations arise from the necessity of developing software and procuring essential equipment like the Leap Motion device and personal computer.

## Conclusions

Engaging in physical exercise through semi-immersive virtual reality utilizing Leap Motion technology emerges as a cost-effective and uncomplicated approach, circumventing the necessity for expensive exercise equipment, particularly robotic systems, and large budgets. Additionally, it boasts portability and ease of application. Prescribed by a physiatrist and overseen by an occupational therapist, this therapeutic regimen involves instructing patients on exercises within a computer-guided virtual environment.

This training method yields increased patient activity, enthusiasm, and adherence to therapy. Leap Motion stands out for its capacity to offer gamified exercises, a feature that holds significant potential for stimulating patient engagement and facilitating the recovery process. By integrating elements of gaming into therapeutic exercises, Leap Motion not only provides a novel and engaging approach to rehabilitation but also taps into the motivational aspects inherent in gamification. This can be particularly beneficial in encouraging patients to actively participate in their rehabilitation, fostering a sense of enjoyment and accomplishment in the process. Unlike tracking technologies involving gloves, which may pose hygiene challenges and potential infection risks, Leap Motion tracking offers contactless hand tracking, potentially representing the future of hand rehabilitation. The programming and control of the application are facilitated through Unity 3D technology, providing a user-friendly environment conducive to game development. The case presented herein suggests avenues for further research and the development of protocols pertaining to the application of this technology in rehabilitation. This exploration aims to contribute to the advancement of knowledge and practices in utilizing semi-immersive virtual reality, specifically integrating leap motion, as an efficacious modality for physical rehabilitation purposes.

## Appendices

For additional information regarding the American Spinal Injury Association (ASIA) Scale or Sollerman Test, please refer to the respective references [19] and [20].

## References

[1] Antal N, Gerta V (2006). Anatomy and physiology of the spinal cord. In: Transplantation of neural tissue into the spinal cord. https://www.ncbi.nlm.nih.gov/books/NBK6229/.

[2] Snell RS (2010). Clinical Neuroanatomy.

[3] National Clinical Guideline Centre (2016). Spinal Injury: Assessment and Initial Management. Spinal Cord Decompression National Institute for Health and Care Excellence (NICE).

[4] Born CT, Gil JA, Goodman AD (2017). Joint contractures resulting from prolonged immobilization: etiology, prevention, and management. J Am Acad Orthop Surg.

[5] (2022). National Guideline Alliance (UK). Physical interventions for people with complex rehabilitations needs after traumatic injury: Rehabilitation after traumatic injury: Evidence review B.1. London: National Institute for Health and Care Excellence. NICE.

[6] Asmaa Saeed (2017). Environments and system types of virtual reality technology in STEM: A survey. Inter Jr Adv Com Sci App.

[7] CREIGHTON CREIGHTON, Ryan Henson (2010). Unity 3D game development by example: A Seat-of-your-pants manual for building fun, groovy little games quickly. Packt Publishing Ltd.

[8] Cortés-Pérez I, Zagalaz-Anula N, Montoro-Cárdenas D, Lomas-Vega R, Obrero-Gaitán E, Osuna-Pérez MC (2021). Leap motion controller video game-based therapy for upper extremity motor recovery in patients with central nervous system diseases. a systematic review with meta-analysis. Sensors (Basel).

[9] Wu J, Yu N, Yu Y (2021). Intraoperative quantitative measurements for bradykinesia evaluation during deep brain stimulation surgery using leap motion controller: a pilot study. Parkinsons Dis.

[10] Butt AH, Rovini E, Dolciotti C, De Petris G, Bongioanni P, Carboncini MC, Cavallo F (2018). Objective and automatic classification of Parkinson disease with Leap Motion controller. Biomed Eng Online.

[11] Garcia-Agundez A, Eickhoff C (2021). Towards objective quantification of hand tremors and bradykinesia using contactless sensors: a systematic review. Front Aging Neurosci.

[12] Cikajlo I, Peterlin Potisk K (2019). Advantages of using 3D virtual reality based training in persons with Parkinson's disease: a parallel study. J Neuroeng Rehabil.

[13] Tarakci E, Arman N, Tarakci D, Kasapcopur O (2020). Leap Motion Controller-based training for upper extremity rehabilitation in children and adolescents with physical disabilities: A randomized controlled trial. J Hand Ther.

[14] Wang ZR, Wang P, Xing L, Mei LP, Zhao J, Zhang T (2017). Leap Motion-based virtual reality training for improving motor functional recovery of upper limbs and neural reorganization in subacute stroke patients. Neural Regen Res.

[15] Alvarez-Lopez F, Maina MF, Saigí-Rubió F (2019). Use of commercial off-the-shelf devices for the detection of manual gestures in surgery: systematic literature review. J Med Internet Res.

[16] Cuesta-Gómez A, Sánchez-Herrera-Baeza P, Oña-Simbaña ED (2020). Effects of virtual reality associated with serious games for upper limb rehabilitation inpatients with multiple sclerosis: randomized controlled trial. J Neuroeng Rehabil.

[17] Arora SP, Naqvi WM (2022). A research protocol on leap motion tracking device: A novel intervention method in distal radial fracture rehabilitation. PLoS One.

[18] Hicks AL, Martin Ginis KA, Pelletier CA, Ditor DS, Foulon B, Wolfe DL (2011). The effects of exercise training on physical capacity, strength, body composition and functional performance among adults with spinal cord injury: a systematic review. Spinal Cord.

[19] Roberts TT, Leonard GR, Cepela DJ (2017). Classifications in brief: American Spinal Injury Association (ASIA) impairment scale. Clin Orthop Relat Res.

[20] Sollerman C, Ejeskär A (1995). Sollerman hand function test. A standardised method and its use in tetraplegic patients. Scand J Plast Reconstr Surg Hand Surg.

